# Interleukin-5 in the Pathophysiology of Severe Asthma

**DOI:** 10.3389/fphys.2019.01514

**Published:** 2019-12-17

**Authors:** Corrado Pelaia, Giovanni Paoletti, Francesca Puggioni, Francesca Racca, Girolamo Pelaia, Giorgio Walter Canonica, Enrico Heffler

**Affiliations:** ^1^Department of Medical and Surgical Sciences, University “Magna Græcia” of Catanzaro, Catanzaro, Italy; ^2^Department of Biomedical Sciences, Humanitas University, Pieve Emanuele, Italy; ^3^Personalized Medicine, Asthma and Allergy, Humanitas Clinical and Research Center, IRCCS, Rozzano, Italy

**Keywords:** IL-5, eosinophils, T2-high asthma, mepolizumab, reslizumab, benralizumab

## Abstract

Interleukin-5 (IL-5) exerts a central pathogenic role in differentiation, recruitment, survival, and degranulation of eosinophils. Indeed, during the last years, significant advances have been made in our understanding of the cellular and molecular mechanisms underlying the powerful actions of IL-5 finalized to the induction, maintenance, and amplification of eosinophilic inflammation. Therefore, IL-5 is a suitable target for add-on biological therapies based on either IL-5 inhibition (mepolizumab, reslizumab) or blockade of its receptor (benralizumab). These modern treatments can result in being definitely beneficial for patients with severe type 2 (T2)-high eosinophilic asthma, refractory to conventional anti-inflammatory drugs such as inhaled and even systemic corticosteroids.

## Introduction

Asthma is a chronic and heterogeneous airway disorder, characterized by recurrent respiratory symptoms including wheezing, cough, and chest tightness, which are caused by usually reversible airflow limitation due to bronchial inflammation and remodeling (Holgate et al., 2015; Pelaia et al., 2015). In particular, many patients with severe asthma express a type 2 (T2)-high phenotype featured by eosinophilic inflammation (Bousquet et al., 1990; Schleich et al., 2014). Indeed, airway eosinophilic infiltration is quite frequent in both allergic and non-allergic asthma, and can also occur in severe and fatal disease (Huber and Koessler, 1922; Houston et al., 1953; Varricchi et al., 2016; Haldar, 2017). T2-high asthma is characterized by accumulation of eosinophils within the airways, where these cells produce and release cytokines, chemokines, growth factors, cytotoxic proteins, and lipid mediators, which together play a relevant role in the pathobiology of bronchial inflammation and remodeling (Bochner and Gleich, 2010). Detection of high eosinophil counts in both peripheral blood and induced sputum is a common feature of T2-high asthma. In this regard, it is noteworthy that airway eosinophilia can occur in more than half asthmatic subjects, and high eosinophil levels are associated with recurrent asthma exacerbations and severe bronchial obstruction (Bousquet et al., 1990).

The most important biological factor responsible for eosinophil differentiation, growth, activation, survival, and recruitment to airways is interleukin-5 (IL-5) (Stirling et al., 2001; Fulkerson and Rothenberg, 2013; Varricchi and Canonica, 2016). Therefore, this cytokine exerts key functions in the pathogenesis of eosinophilic asthma, which is often therapeutically responsive to corticosteroids because of its effective ability to induce eosinophil apoptosis (Zhang et al., 2000). However, severe eosinophilic asthma may be resistant to both inhaled and systemic corticosteroids, also because of an excessive bronchial amount of IL-5, which can thereby overcome the pro-apoptotic effects of these drugs on eosinophils (Dunican and Fahy, 2017). Hence, patients with severe T2-high eosinophilic asthma, refractory to corticosteroids, may experience an inadequate control of respiratory symptoms and frequent disease exacerbations, thus being characterized by relevant unmet needs. Moreover, in these subjects, IL-5-dependent eosinophilia can also contribute to the development of clinically significant comorbidities such as chronic rhinosinusitis with nasal polyps. Indeed, these upper airway disorders originate from cellular and molecular mechanisms, which appear to be very similar to those underlying type 2 inflammation in asthma (Heffler et al., 2018, 2019; Ahern and Cervin, 2019).

For all such reasons, in severe T2-high asthma, IL-5 represents a pivotal pathogenic factor and a highly valuable target for add-on biological therapies of corticosteroid-resistant, difficult-to-treat eosinophilic phenotypes (Varricchi and Canonica, 2016; Brussino et al., 2018). In particular, several monoclonal antibodies have been developed against either IL-5 (mepolizumab, reslizumab) or its receptor (benralizumab), thereby making it possible to break down the main pathobiological pathway implicated in eosinophilic asthma (Egan et al., 1999; Gnanakumaran and Babu, 2003; Kolbeck et al., 2010; Pelaia et al., 2016; Bagnasco et al., 2017; Pelaia et al., 2017; Varricchi et al., 2017a,b; Bagnasco et al., 2018a,b; Pelaia et al., 2018a,b,c, 2019).

Taking together the above considerations, it is very clear that IL-5 plays a central role as the most important pathogenic mediator responsible for eosinophilic asthma, as well as a crucial therapeutic target for anti-asthma biological treatments. Therefore, the aim of the present review article is to discuss the pathobiological interactions between IL-5 and T2-high eosinophilic asthma, the mechanism of action of IL-5, and the relevance of both this cytokine and its receptor as targets of selective anti-eosinophil monoclonal antibodies.

## IL-5 and Eosinophilic Asthma

**Figure 1:**
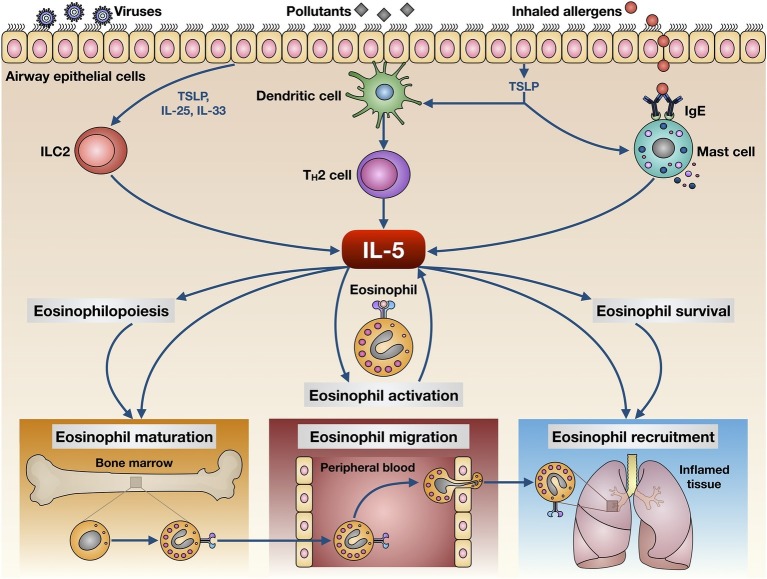
Biological actions exerted by IL-5 on eosinophils. IL-5 is produced by several cellular elements, including Th2 lymphocytes, group 2 innate lymphoid cells (ILC2), mast cells, and eosinophils. These cells release IL-5 upon activation triggered by multiple environmental stimuli such as inhaled allergens, respiratory viruses, and airborne pollutants. IL-5 exerts pleiotropic effects on eosinophils, thereby promoting their maturation, activation, survival, migration from bloodstream, and recruitment to airways.

The main cellular sources of IL-5 include T helper-2 (Th2) lymphocytes and group 2 innate lymphoid cells (ILC2) (Figure 1; Woodruff et al., 2009; Brusselle et al., 2013; Walker et al., 2013; Smith et al., 2016; Yanagibashi et al., 2017). Th2 cells produce and secrete IL-5 upon a complex activation process triggered by inhaled allergens and driven by dendritic cells (Lambrecht et al., 2019). In this regard, the presence of interleukin-4 (IL-4) is essential, because of its requirement for Th2 cell commitment and activation *via* stimulation of key transcription factors such as STAT6 and GATA3 (Lambrecht and Hammad, 2015). IL-5 release from ILC2 is dependent on GATA3 activation induced by epithelial innate cytokines including IL-25, IL-33, and especially thymic stromal lymphopoietin (TSLP) (Figure 1; Lambrecht and Hammad, 2015). In addition to ILC2 and Th2 cells, other cellular sources of IL-5 include invariant natural killer (NK) T cells, mast cells, and eosinophils themselves (Figure 1; Shakoory et al., 2004; Sakuishi et al., 2007; Hogan et al., 2008). In particular, by releasing IL-5 activated mast cells implement a bidirectional cross-talk with eosinophils (Galdiero et al., 2017). Such functional interactions between mast cells and eosinophils, also supported by physical contacts involving these two cell types, harbor the so-called “allergic effector unit” (Minai-Fleminger et al., 2010; Galdiero et al., 2017).

IL-5 is a powerful pro-inflammatory cytokine that is responsible for maturation, proliferation, activation, and migration of eosinophils (Figure 1). The close pathogenic link between IL-5 and eosinophilic inflammation has been clearly demonstrated using both animal and human experimental models of asthma (Greenfeder et al., 2001). In particular, IL-5 is responsible for airway eosinophilia and bronchial hyperresponsiveness induced by allergen challenge in sensitized guinea pigs (Mauser et al., 1993). Moreover, in the lungs of these animals, an eosinophilic inflammatory response can be experimentally evoked by recombinant human IL-5 (Lilly et al., 1996). Similar to guinea pigs, upon allergen challenge, an IL-5-dependent influx of eosinophils was also detected in bronchoalveolar lavage fluid (BALF) and lung tissue of sensitized mice (Kung et al., 1994, 1995). Such results have been further corroborated by demonstrating that bronchial eosinophilia and airway hyperresponsiveness, induced by multiple allergen challenges, were abrogated in sensitized IL-5-deficient mice (Foster et al., 1996; Kopf et al., 1996). In experimental monkey models of asthma, IL-5 was capable of inducing bronchial eosinophilia and the consequent airway hyperresponsiveness (Mauser et al., 1995). Furthermore, it has been shown in both rabbits and humans that delivery of recombinant IL-5 to airway smooth muscle enhanced the contractile response to acetylcholine (Hakonarson et al., 1999), and this effect was probably mediated by the release of eosinophil granule proteins (Elbon et al., 1995). In atopic patients experiencing both early and late asthmatic reactions, the bone marrow responds to antigen challenge by enhancing the production of eosinophils, which resulted in being associated with an increase in IL-5 mRNA levels (Wood et al., 2002). In addition, IL-5 prolonged eosinophil survival in allergen-challenged atopic asthmatics (Ohnishi et al., 1993).

In allergic asthmatic subjects, the eosinophilopoietic actions of IL-5 take place in both bone marrow and bronchial mucosa (“*in situ* eosinophilopoiesis”), where this cytokine promotes eosinophil differentiation and maturation from CD34+ hematopoietic progenitor cells (Wood et al., 2002; Dorman et al., 2004; Bhalla et al., 2018). In fact, elevated IL-5 levels and high cell counts of eosinophil progenitors and mature eosinophils can be found in induced sputum from patients with allergic asthma (Dorman et al., 2004). Furthermore, in comparison to both healthy controls and subjects with mild asthma, higher serum IL-5 concentrations were detected in patients with severe disease (Greenfeder et al., 2001). IL-5 synergizes with eotaxins, thus contributing to recruit eosinophils to asthmatic airways (Fulkerson and Rothenberg, 2013). Indeed, high levels of IL-5 and eotaxins were found in induced sputum from patients experiencing acute asthma exacerbations (Park et al., 2003). A synergic action is also exerted by IL-5 in conjunction with IL-18 (Kandikattu et al., 2019). In particular, concomitant increases of serum levels of IL-5 and IL-18 were found in patients with asthma, and the concentrations of these two cytokines correlated with disease exacerbations (Kandikattu et al., 2019). IL-5 and IL-18 strongly cooperate to induce eosinophil development and functional activation. IL-5 also inhibits eosinophil apoptosis, and sputum IL-5 levels were reported to be negatively correlated with apoptotic eosinophils in subjects with either asthma exacerbations or stable disease (Xu et al., 2007; Ilmarinen et al., 2014). Moreover, in T2-high asthma IL-5 induces eosinophil adhesion to and migration in the extracellular matrix by favoring the interaction of eosinophils with periostin, a matricellular protein whose enhanced expression is associated with eosinophil trafficking toward bronchi (Johansson, 2017). IL-5 is also involved in the pathobiology of late-onset, non-allergic eosinophilic asthma (Brusselle et al., 2013). In this case, ILC2 and not Th2 lymphocytes are mainly responsible for IL-5 production (Walker et al., 2013). Differently from blood and airway pro-inflammatory eosinophils, the lung resident subsets of homeostatic anti-inflammatory and anti-allergic eosinophils seem to be partially independent from IL-5, at least in mice (Marichal et al., 2017).

With regard to the pathobiology of asthma, in addition to promoting the development and amplification of eosinophilic inflammation, IL-5 is also implicated in the induction of airway remodeling (Kay et al., 2004). Indeed, in murine models of asthma, it has been shown that IL-5 gene deletion was associated with a parallel suppression of both lung eosinophilia and bronchial remodeling (Cho et al., 2004). On the other hand, IL-5 transgenic mice were reported to be characterized by an enhanced airway fibrotic response to repeated allergen challenges (Tanaka et al., 2004). The results of these animal studies have been further corroborated by examining the bronchial biopsies taken from asthmatic patients treated with an anti-IL-5 monoclonal antibody (Flood-Page et al., 2003). In particular, it was demonstrated through confocal microscopy that anti-IL-5 treatment decreased the thickness of reticular basement membrane by reducing the deposition of extracellular matrix proteins such as procollagen III, tenascin, and lumican (Flood-Page et al., 2003).

## IL-5: Mechanism of Action

**Figure 2:**
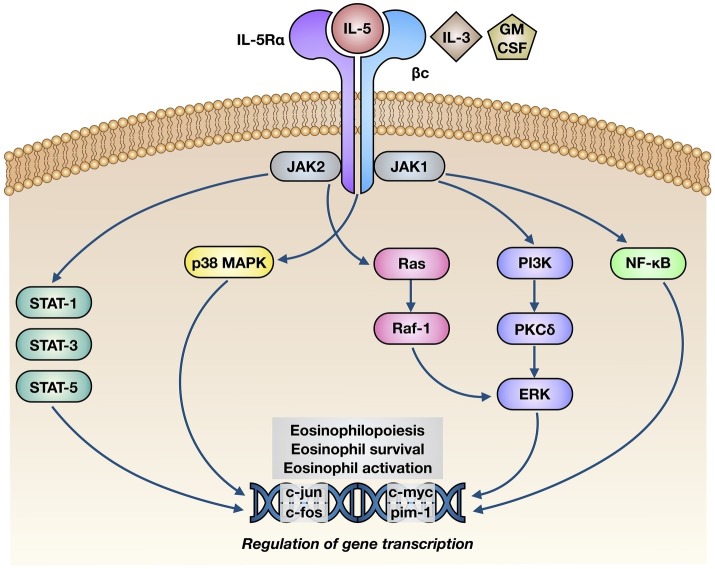
Molecular mechanisms of action underlying the effects of IL-5 on eosinophils. IL-5 binds to the α subunit of IL-5 receptor (IL-5Rα), thereby inducing its interaction with βc subunits and the following activation of a complex intracellular signaling network, consisting of JAK1/2-STAT1/3/5 modules, p38 and ERK MAP kinases, and NF-κB transcription factor. The consequent stimulation of specific target genes leads to eosinophil maturation, survival, and activation.

The biological effects of IL-5 are mediated by its selective interaction with the IL-5 receptor (IL-5R), consisting of a specific α subunit (IL-5Rα) and a non-specific βc heterodimer, which can be recognized also by interleukin-3 (IL-3) and granulocyte-macrophage colony stimulating factor (GM-CSF) (Figure 2; Rossjohn et al., 2000; Murphy and Young, 2006). IL-5 binds as a homodimeric protein to IL-5Rα, which is highly expressed on eosinophil surface (Varricchi et al., 2016), thus recruiting the βc dimer and inducing the assembly of the IL-5/IL-5Rα/βc ternary complex (Broughton et al., 2012). When IL-5 is absent, IL-5Rα is complexed with the intracellular tyrosine kinase Janus kinase (JAK)2, whereas the βc subunit is associated with JAK1 (Kouro and Takatsu, 2009). When IL-5 is present, it binds to IL-5Rα and drives the constitution of a functional IL-5Rα/βc receptor complex, which is responsible for the activation of an intricate network of signaling pathways (Figure 2; Johanson et al., 1995; Ishino et al., 2008; Molfino et al., 2012). In particular, binding of IL-5 to IL-5Rα sequentially activates JAK2 and signal transducers and activators of transcription (STAT)1, 3, and 5, which in turn stimulate the transcriptional functions of many genes involved in eosinophil proliferation, including pim-1 and cyclin D3 (Pazdrak et al., 1995; Stout et al., 2004). Moreover, JAK2 is engaged in active cooperation with Lyn and Raf-1 kinases, and such functional interactions lead to inhibition of eosinophil apoptosis (Pazdrak et al., 1998); the inhibitory effect of IL-5 on eosinophil apoptosis is also mediated by NF-κB-dependent induction of the anti-apoptotic protein Bcl-xL (Schwartz et al., 2015; Amruta and Kandikattu, 2018). Raf-1 also stimulates eosinophil degranulation (Pazdrak et al., 1998).

Other signal transduction modules activated by IL-5 include further intracellular kinases such as phosphoinositide 3-kinase (PI3K) and mitogen-activated protein kinases (MAPK) (Figure 2). In particular, *via* activation of extracellular signal-regulated kinases (ERK)1/2 and protein kinase C (PKC), PI3K mediates IL-5-induced interaction of eosinophils with intercellular adhesion molecule-1 (ICAM-1) (Sano et al., 2005). Ras-Raf-1-mediated activation of the ERK subfamily of MAPK drives c-fos gene transcription, which is involved in several eosinophil functions including cell maturation, survival, and proliferation, as well as stimulation of the production of the powerful eosinophil chemoattractant leukotriene C4 (Adachi and Alam, 1998; Bates et al., 2000; Pelaia et al., 2005; Takatsu and Nakajima, 2008; Thompson-Souza et al., 2017). Furthermore, through a NF-κB-dependent mechanism, p38 MAPK up-regulates eosinophil biosynthesis of pro-inflammatory cytokines, and also stimulates eosinophil recruitment within the context of allergic inflammatory responses (Adachi et al., 2000; Ip et al., 2005; Pelaia et al., 2005).

Therefore, because of the pivotal role played by IL-5 in the pathophysiology of T2-high asthma, this cytokine and its receptor represent key molecular targets for current biological therapies aimed to improve the control of severe and difficult-to-treat eosinophilic disease (Varricchi et al., 2016; Bagnasco et al., 2018a,b; McGregor et al., 2019; Siddiqui et al., 2019).

## IL-5 and Its Receptor: Molecular Targets for Biological Therapies of Severe Asthma

**Figure 3:**
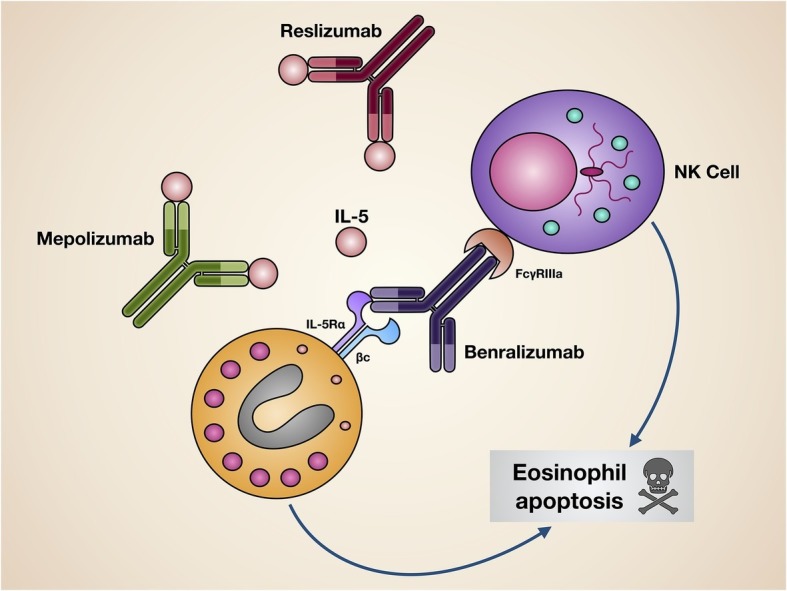
Mechanisms of action of biological drugs targeting IL-5 or its receptor. Mepolizumab and reslizumab interact with IL-5, thus inhibiting its biological effects on eosinophils. Benralizumab blocks *via* its Fab fragments IL-5Rα, thereby neutralizing IL-5 bioactivity. Moreover, through its Fc constant region benralizumab binds to the FcγIIIRa receptor expressed by natural killer cells, enabling them to induce eosinophil apoptosis.

In clinical practice, three monoclonal antibodies, namely mepolizumab, reslizumab, and benralizumab, are currently available, which make it possible to effectively interfere with the pathogenic IL-5/IL-5R pro-eosinophilic axis. While mepolizumab and reslizumab are selective IL-5 inhibitors, benralizumab is an IL-5 receptor antagonist (Figure 3).

Mepolizumab (SB-240563) is a humanized IgG1/k monoclonal antibody that specifically binds with high affinity to IL-5, thereby impeding its linkage to IL-5Rα (Figure 3; Gnanakumaran and Babu, 2003; Walsh, 2015; Fainardi et al., 2016; Varricchi et al., 2017a). In particular, mepolizumab was developed by incorporating specific murine antibody fragments targeting human IL-5 into a human IgG1 heavy chain (Hart et al., 2001). The relevant benefits induced by mepolizumab in patients with severe refractory eosinophilic asthma have been clearly documented by many randomized controlled trials (RCT) (Pelaia et al., 2017; Varricchi et al., 2017a). Initially, the efficacy of mepolizumab was demonstrated by Nair et al. and Haldar et al. in a few patients with severe eosinophilic asthma experiencing frequent disease exacerbations (Haldar et al., 2009; Nair et al., 2009), These two seminal studies were the first ones to show that mepolizumab was able to significantly decrease asthma exacerbations, and this effect was concomitant with a sharp reduction of both sputum and blood eosinophils (Haldar et al., 2009; Nair et al., 2009). In addition to such results, using chest CT (computed tomography) scan, Haldar et al. also documented that mepolizumab decreased both thickness and total area of bronchial walls (Haldar et al., 2009). These observations corroborated previous findings published by Flood-Page et al., who reported that mepolizumab was capable of reducing the amount of extracellular matrix proteins deposited within the sub-epithelial reticular basement membrane of airway mucosa; this effect was associated with decreased BALF concentrations of transforming growth factor-β1 (TGF-β1) (Flood-Page et al., 2003). Therefore, it can be inferred that the potential anti-remodeling effect of mepolizumab was probably a consequence of the depleting action exerted on eosinophils, which are important cellular sources of TGF-β1, a prominent growth factor involved in the pathobiology of the airway structural changes occurring in asthma (Makinde et al., 2007).

Later, Pavord et al. performed the phase 2b/3 DREAM (Dose Ranging Efficacy And safety with Mepolizumab in severe asthma) trial, thus confirming, in a much larger study population, that mepolizumab lowered sputum and blood eosinophil levels, and also significantly decreased the asthma exacerbation rate (Pavord et al., 2012). Subsequently, two further studies named MENSA (MEpolizumab as adjunctive therapy iN patients with Severe Asthma) and SIRIUS (SteroId ReductIon with mepolizUmab Study) were conducted by Ortega et al. and Bel et al., respectively. Both trials demonstrated that, in patients with severe eosinophilic asthma, mepolizumab decreased the number of asthma exacerbations, improved symptom control and quality of life, and also induced a slight FEV_1_ (forced expiratory volume in 1 s) increase (Bel et al., 2014; Ortega et al., 2014), Moreover, the SIRIUS trial provided convincing evidence about the oral corticosteroid-sparing action of mepolizumab, consisting of a 50% decrease in prednisone intake (Bel et al., 2014). More recently, the phase IIIb MUSCA study, performed by Chupp et al., confirmed the ability of mepolizumab to improve health-related quality of life (Chupp et al., 2017). All these studies also showed that mepolizumab is characterized by a very good safety and tolerability profile. The main RCT referring to mepolizumab have been summarized in Table 1. In addition to RCT, mepolizumab is also undergoing evaluation within the context of real-life studies carried out in daily clinical practice. In this regard, preliminary data suggest that in a real-world setting mepolizumab can result in being even more effective than in RCT, and such findings might depend on the higher blood eosinophil counts characterizing real-life patients when compared to asthmatics enrolled in RCT (Pelaia et al., 2018a; Bagnasco et al., 2018b).

**Table 1:** Mepolizumab: main randomized clinical trials.

Authors	Inclusion criteria	*N*	Main results
Flood-Page et al. (2003)	Mild atopic asthmatics	11	↓ Blood and BALF eosinophils = FEV_1_, = PEF, = airway hyperresponsiveness
Haldar et al. (2009)	Eosinophilic asthma	61	↓ Blood and sputum eosinophils = FEV_1_, = FeNO, = airway hyperresponsiveness ↓ Exacerbations ↑ QoL
Nair et al. (2009)	Prednisone-dependent eosinophilic asthma	9	↓ Blood and sputum eosinophils ↓ Exacerbations
Pavord et al. (2012)	Severe eosinophilic asthma	462	↓ Blood and sputum eosinophils = FEV_1_, = FeNO, = AQLQ, = ACQ ↓ Exacerbations
Ortega et al. (2014)	Severe eosinophilic asthma	385	↑ FEV_1_ ↓ Exacerbations, ↓ Hospitalizations ACQ-5 and SGRQ improvement
Bel et al. (2014)	Severe eosinophilic asthma	135	↓ Blood and sputum eosinophils ↓ Exacerbations, ↓ OCS intake ACQ-5 improvement
Chupp et al. (2017)	Severe eosinophilic asthma	274	↑ FEV_1_, ↑ FEF_25–75_ ACQ-5 and SGRQ improvement

*BALF, Bronchoalveolar lavage fluid; FEV_1_, Forced expiratory volume in 1 s; PEF, Peak expiratory flow; FeNO, Exhaled fraction of nitric oxide; QoL, Health-related quality of life; AQLQ, Asthma quality of life questionnaire; ACQ, Asthma control questionnaire; SGRQ, St. George’s Respiratory Questionnaire; OCS, Oral corticosteroids; FEF_25–75_, Forced expiratory flow at 25–75% of forced vital capacity*.

Reslizumab (SCH55700) is a humanized IgG4/κ monoclonal antibody which includes in its structure the complementarity-determining regions of the rat monoclonal IgG2a antibody JES1-39D10, that specifically interact with the epitope encompassing amino acids 89–92 of human IL-5, thereby preventing its binding to IL-5Rα (Figure 3; Zhang et al., 1999). In regard to add-on biological therapy of severe eosinophilic asthma, the efficacy and safety of reslizumab have been evaluated in several RCT (Pelaia et al., 2016; Varricchi et al., 2017b). The first phase 2 study was performed by Kips et al., who showed that reslizumab lowered blood and sputum eosinophil counts, and also induced a transient FEV_1_ increase (Kips et al., 2003). A subsequent, larger phase 2 trial, carried out by Castro et al., demonstrated that reslizumab significantly increased FEV_1_, and also elicited a non-significant trend toward a better asthma control, especially in highly eosinophilic patients with concomitant nasal polyposis (Castro et al., 2011), Later, two phase 3 studies were conducted by Castro et al., who demonstrated the effectiveness of reslizumab in decreasing by 50–59% the annual rate of asthma exacerbations in severe asthmatics with blood eosinophil counts >400 cells/ml (Castro et al., 2015); reslizumab also improved asthma symptom control and enhanced FEV_1_ (Castro et al., 2015). The beneficial effects of reslizumab on lung function were further confirmed by another phase 3 trial carried out by Bjermer et al., who reported that reslizumab not only increased FEV_1_, but also improved airflow limitation at level of peripheral airways, as shown by significant increases in FEF_25–75_ (forced expiratory flow at 25–75% of forced vital capacity) (Bjermer et al., 2016). More recently, an additional phase 3 trial performed by Brusselle et al. highlighted that reslizumab was able to reduce asthma exacerbations and improve lung function, especially in patients with eosinophilic late-onset asthma (Brusselle et al., 2017). Taken together, the results of the above studies evidenced a good safety and tolerability profile of reslizumab. The main RCT referring to reslizumab have been summarized in Table 2.

**Table 2:** Reslizumab: main randomized clinical trials.

Authors	Inclusion criteria	*N*	Main results
Kips et al. (2003)	Severe asthmatics	18	↓ Blood and sputum eosinophils Transient FEV_1_ increase
Castro et al. (2011)	Poorly controlled eosinophilic asthma	61	↓ Blood and sputum eosinophils ↑ FEV_1_, ↑ FVC ACQ-5 improvement
Castro et al. (2015)	Severe eosinophilic asthma	953	↓ Blood eosinophils ↑ FEV_1_ ↓ Exacerbations AQLQ, ACQ-7, ASUI improvement
Bjermer et al. (2016)	Severe eosinophilic asthma	315	↓ Blood eosinophils ↑ FEV_1_, ↑ FEF_25–75_ ACQ-5, ACQ-6, AQLQ, ASUI improvement
Brusselle et al. (2017)	Severe eosinophilic asthma	477	↓ Exacerbations ↑ FEV_1_

*FEV_1_, Forced expiratory volume in 1 s; FVC, forced vital capacity; PEF, Peak expiratory flow; ACQ, Asthma control questionnaire; AQLQ, Asthma quality of life questionnaire; ASUI, Asthma symptom utility index; FEF_25–75_, forced expiratory flow at 25–75% of forced vital capacity*.

Benralizumab (MEDI-563) is a humanized afucosylated IgG1/κ monoclonal antibody, developed *via* hybridoma technology, whose Fab fragments contain murine amino acid sequences which selectively recognize the isoleucine-61 residue of the domain 1 of human IL-5Rα, located near IL-5 binding site (Ishino et al., 2004; Koike et al., 2009; Kolbeck et al., 2010). As a consequence, the interaction of benralizumab with its recognition site on IL-5Rα impedes IL-5 binding to target cells (Figure 3), thus preventing hetero-dimerization of IL-5Rα and βc subunits, as well as the subsequent activation of IL-5-dependent signaling pathways. Furthermore, through the constant Fc region benralizumab binds to the FcγRIIIa membrane receptor expressed by natural killer cells (Figure 3), which upon FcγRIIIa activation release the pro-apoptotic proteins granzyme B and perforin, responsible for eosinophil apoptosis implemented *via* antibody-dependent cell-mediated cytotoxicity (ADCC), a mechanism which is markedly amplified by afucosylation (Shields et al., 2002; Ghazi et al., 2012).

Several phase 3 RCT have recently shown that, as add-on treatment of severe eosinophilic asthma, benralizumab is characterized by an excellent pattern of efficacy, safety, and tolerability (Pelaia et al., 2018b,c; Gonzalez et al., 2019). In particular, CALIMA and SIROCCO trials showed that benralizumab significantly decreased the annual rate of severe eosinophilic exacerbations of asthma, and also improved asthma symptom control and enhanced FEV_1_ (Bleecker et al., 2016; FitzGerald et al., 2016). Benralizumab-induced improvement in lung function was also confirmed by BISE study (Ferguson et al., 2017). In addition, the ZONDA study demonstrated that benralizumab was able to significantly lower the daily intake of oral corticosteroids (Nair et al., 2017). Moreover, the BORA trial showed that a long-term use of benralizumab was associated with a very good safety and tolerability profile (Busse et al., 2019). Furthermore, it is noteworthy that benralizumab appears to be very effective in both allergic and non-allergic severe asthma (Chipps et al., 2018). The main RCT referring to benralizumab have been summarized in Table 3. The latter findings have also been recently confirmed by preliminary real-life observations, which suggest that in daily clinical practice the therapeutic actions of benralizumab may result in being even more rapid and effective with respect to RCT (Pelaia et al., 2019).

**Table 3:** Benralizumab: main randomized clinical trials.

Authors	Inclusion criteria	*N*	Main results
Bleecker et al. (2016)	Severe asthma	797	↑ FEV_1_ ↓ Exacerbations ACQ-6 and AQLQ improvement
FitzGerald et al. (2016)	Severe eosinophilic asthma	866	↑ FEV_1_ ↓ Exacerbations ACQ-6 and AQLQ improvement
Ferguson et al. (2017)	Severe eosinophilic asthma	106	↓ Blood eosinophils ↑ FEV_1_ = ACQ-6, = AQLQ
Nair et al. (2017)	Severe eosinophilic asthma	145	↓ Exacerbations ↓ OCS intake
Busse et al. (2019)	Severe eosinophilic asthma	1,576	Long-term safety and tolerability

*FEV_1_, Forced expiratory volume in 1 s; ACQ, Asthma control questionnaire; AQLQ, Asthma quality of life questionnaire; OCS, Oral corticosteroids*.

In regard to IL-5 receptor blockade finalized to the treatment of eosinophilic asthma, a potential alternative approach to the use of monoclonal antibodies can be represented by the development of small molecule antagonists (Uings and McKinnon, 2002). Within such a context, an isothiazolone compound was identified, which appeared to be able to selectively interfere with IL-5/IL-5R interaction (Devos et al., 1994; Uings and McKinnon, 2002). However, to our knowledge this small molecule IL-5R antagonist has not yet reached the stage of clinical investigation.

## Concluding Remarks

**Figure 4:**
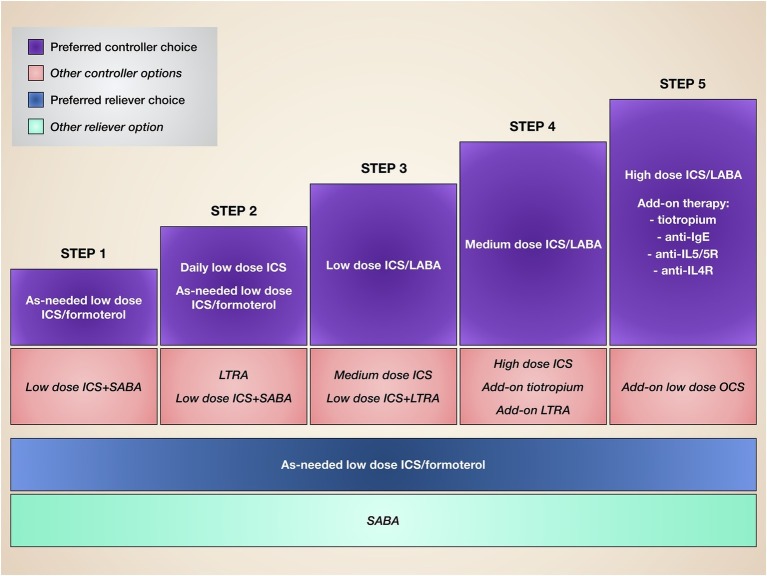
Stepwise therapy of asthma. Current asthma treatment is based on a stepwise approach, consisting of progressive therapeutic increases until disease control is achieved.

Our very strong awareness of the pivotal pathobiological role played by IL-5 in T2-high eosinophilic asthma makes it critical to carefully characterize asthmatic patients on the basis of their inflammatory substrate, as well as in consideration of the clinical and functional responses to standard treatments. Indeed, the most relevant unmet needs are experienced by both allergic and non-allergic asthmatics who are not well controlled by corticosteroids, also because of the prominent pro-eosinophilic action of IL-5, which probably overwhelms the potential efficacy of conventional anti-inflammatory drugs. Therefore, under such circumstances, IL-5 and its receptor may represent valuable therapeutic targets. In this regard, several RCT and some preliminary real-life studies have clearly shown that mepolizumab, reslizumab, and benralizumab are safe and effective as add-on biological therapies for patients with difficult-to-treat eosinophilic asthma. Indeed, such biologics are currently included within the step 5 of GINA (Global Initiative for Asthma) guidelines (Figure 4; Global Initiative for Asthma, 2019). Therefore, the only limitation of these monoclonal antibodies depends on their high cost (Anderson and Szefler, 2019). Although the use of anti-eosinophilic biological treatments for severe asthma can significantly decrease the intake of oral corticosteroids, the number of emergency visits and hospitalizations, as well as the loss of work- and school-days, and their cost-effectiveness should be improved by price reductions eventually provided by manufacturers (Anderson and Szefler, 2019). Hopefully, lower costs of mepolizumab, reslizumab, and benralizumab could make these drugs more affordable by health care systems of economically weak countries.

## Author Contributions

All authors listed have made a substantial, direct and intellectual contribution to the work, and approved it for publication.

### Conflict of Interest

The authors declare that the research was conducted in the absence of any commercial or financial relationships that could be construed as a potential conflict of interest.
